# Gonadal Dysgenesis 46, XX Associated with Mayer-Rokitansky-Kuster-Hauser Syndrome: One Case Report

**DOI:** 10.1155/2010/847370

**Published:** 2010-12-29

**Authors:** N. Bousfiha, S. Errarhay, H. Saadi, K. Ouldim, C. Bouchikhi, A. Banani

**Affiliations:** ^1^Department of Gynecology Obstetric I, Teaching Hospital Hassan II, Fez 30000, Morocco; ^2^Department of Medical Genetic and Oncogenetic, Teaching Hospital Hassan II, Fez 30000, Morocco

## Abstract

*Introduction*. The association of gonadal dysgenesis and Mayer-Rokitansky-Kuster-Hauser syndrome is very rare and appears to be coincidental, independent of chromosomal anomalies. *Case Report*. We report the case of a 19-year-old woman who presented primary amenorrhea and impuberism. The endocrine study revealed hypergonadotrophic hypogonadism. The karyotype was normal, 46XX. No chromosome Y was detected at the FISH analysis. Internal genitalia could not be identified on the pelvic ultrasound and pelvic MRI. Laparoscopy was undertaken and revealed concomitant ovarian dysgenesis and Mayer-Rokitansky-Kuster-Hauser syndrome. There were no other morphological malformations. *Conclusion*. The pathogenesis of the association of gonadal dysgenesis and Mayer Rokitansky kuster hauser syndrome is still mysterious. The treatment is based essentially on hormone substitution therapy. The fertility prognosis is unfortunately compromised.

## 1. Introduction

Gonadal dysgenesis with female phenotype is defined as the absence or insufficient development of the ovaries. It causes primary amenorrhea with variable hypogonadism or impuberism, depending on the degree of gonadal development. The karyotype can be 46,XX; 45,X0; 46,XY or mosaïcism 45,X/46,XX; 45,X/46,X,del(X)(p22.2); 46,X,i(Xq) [1–3]. The Mayer-Rokitansky-Kuster-Hauser Syndrome (MRKHS) is a specific type of mullerian duct malformation characterized by congenital absence or hypoplasia of uterus and upper two thirds of the vagina in both phenotypically and karyotypically normal females with functional ovaries [4]. It is the second most common cause of primary amenorrhea.

 An association between these two conditions is very exceptional and appears to be coincidental, independent of chromosomal anomalies.

## 2. Case Report

 We report the case of a 19-year-old Moroccan woman who presented primary amenorrhea and impuberism.

Her height was 168 cm, weight 54 Kg, and blood pressure 120/70 mm Hg. No other affected members family were detected. Physical examination revealed no abnormalities. Pubic, axillary hair growth and breast development were scored, respectively, Tanner stages II and III. The patient had no evidence of facial dysmorphism, webbing of the neck, or skeletal abnormalities. A 1 cm vagina ending in a blind pouch was found on gynecological examination. Bimanual pelvic and rectovaginal examination revealed no evidence of a cervix or uterus. 

**Figure 1:**
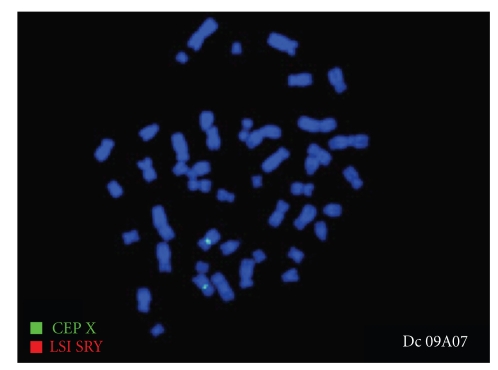
Fluorescence *in situ* hybridization FISH: the chromosomes can be seen in blue LSI SRY: spectrum orange/CEP X: spectrum green. Presence of 2 green spots. No orange spot was detected.

**Figure 2:**
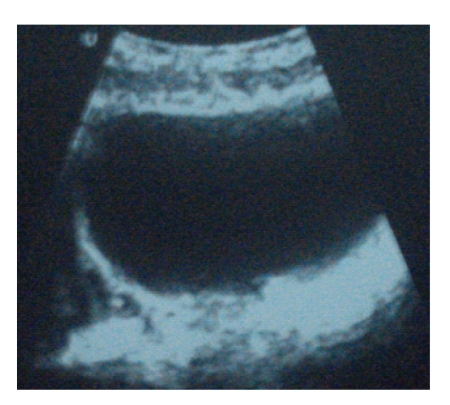
Ultrasound exam: no uterus identified behind the bladder.

**Figure 3:**
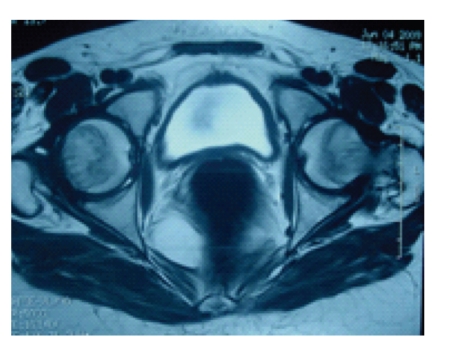
Magnetic resonance imaging (MRI): axial plane cut showing bladder and rectum without interposition of uterus.

**Figure 4:**
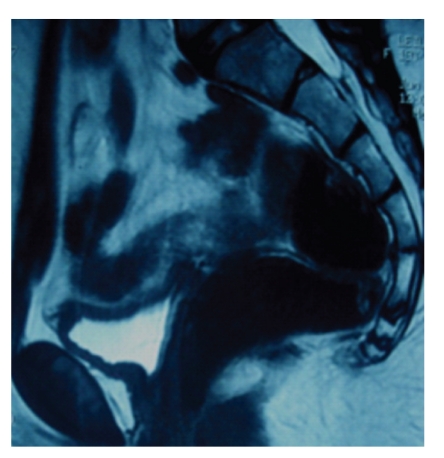
MRI examination sagittal plane cut: absence of uterus and ovaries.

An endocrine study including pituitary, ovarian, and thyroid evaluation was performed and reveled hypergonadotrophic hypogonadism (Luteinizing Hormone: 16.07 UI/L, Follicle Stimulating Hormone: 42.48 UI/L, Oestradiol: 24.24 UI/L) with normal level of prolactin and Thyroid Stimulating Hormone. The karyotype was 46XX (Figure 1), and no Y chromosome material was detected in fluorescence in situ hybridization (FISH). Internal genitalia could not be identified on the pelvic ultrasound (Figure 2) nore on the IRM (Figures 3 and 4). Laparoscopy was undertaken and revealed concomitant ovarian dysgenesis and Mayer-Rokitansky-Kuster-Hauser syndrome. 

Bone study, pelvic ultrasound, and laparoscopic study were ordered to evaluate the associated genitourinary and skeletal anomalies. There were no other morphological malformations.

We conclude to an association of MRKH with ovarian agenesis. Hormonal substitution by estrogen and progesterone was then undertaken.

## 3. Discussion

The ovaries are embryologically derived from 3 sources: mesodermal epithelium (lining the posterior or abdominal wall), underlying mesenchyme (embryonic connective tissue), and primordial germ cells. The epithelium and mesenchyme proliferate to produce the genital (gonadal) ridge. The primordial germ cells migrate along the dorsal mesentery of the hindgut to the genital ridges and enter the underlying mesenchyme. If the primordial germ cells do not form or migrate into the gonadal area, an ovary will not develop. 

 The mullerian (paramesonephric) ducts develop lateral to the gonads and play an essential role in the development of uterine tubes, uterus, superior part of the vagina and broad ligaments. Estrogens may influence development of the paramesonephric system. Absence of mullerian-inhibiting substance (antimullerian hormone) is also essential for its development. Mutations of the gene encoding the antimullerian hormone receptor and the lack of estrogen receptors during embryonic development have been hypothesized to cause MRKH syndrome [2]. 

An undifferentiated gonad may produce antimullerian hormone in earlier embryologic period. But this hypothesis cannot be valuable without the presence of the chromosome Y [5]. The FISH analysis of karyotype of our patient demonstrated the absence of chromosome Y.

 Few cases of association of ovarian dysgenesis and MRKH syndrome are reported in the literature indeed [1, 2, 6–10]. 

Unfortunately these two conditions compromise the prognosis of fertility of young patients. 

Hormone substitution therapy remains the only therapeutic option. It is aimed at triggering the development of secondary sexual characters and prevent osteoporosis. There remains the unsolved problem of infertility [6].

## 4. Conclusion

The association of gonadal dysgenesis and Mayer Rokitansky kuster hauser syndrome is very rare and appears to be coincidental, independent of chromosomal anomalies. These two conditions compromise the fertility both in the mechanic and hormonal plane. Actually the treatment is based on hormone substitution therapy. The aim of the treatment is to trigger the development of secondary sexual characters and prevent osteoporosis.
